# Optimizing Delivery Timing in Pregnant Patients With Chronic Hypertension at Term

**DOI:** 10.1097/og9.0000000000000050

**Published:** 2024-12-05

**Authors:** Ira Hamilton, James Liu, Labeena Wajahat, Emily A. DeFranco, Robert Rossi

**Affiliations:** Division of Maternal-Fetal Medicine, Department of Obstetrics and Gynecology, University of Cincinnati College of Medicine, Cincinnati, Ohio.

## Abstract

**OBJECTIVE:**

To estimate the optimal timing of delivery among pregnant patients with chronic hypertension at term.

**METHODS:**

We performed a population-based retrospective cohort study including all nonanomalous singleton term (37–42 weeks of gestation) births in the United States from 2014 to 2018 among patients with chronic hypertension, excluding those with superimposed preeclampsia, eclampsia, and pregestational diabetes. The rates of stillbirth, infant death (within 1 year of life), and neonatal morbidity were compared at each week of term pregnancy. *Neonatal morbidity* was defined as a composite of neonatal intensive care unit admission, ventilation for 6 hours or longer, low 5-minute Apgar score (3 or lower), and seizures. To estimate the optimal delivery timing among pregnant patients with chronic hypertension, the risk of delivery at each week (ie, the rate of infant death or neonatal morbidity) was compared with the risk of expectant management (ie, the rate of stillbirth over that week plus rate of infant death or neonatal morbidity in the subsequent week) for an additional week. Subgroup analyses were performed for pregnancies complicated by fetal growth restriction and among non-Hispanic Black patients with chronic hypertension.

**RESULTS:**

Among pregnant patients with chronic hypertension at term (N=227,977), the rate of stillbirth (per 10,000 ongoing pregnancies) overall increased with gestational age and was lowest at 38 weeks (6.5, 95% CI, 5.4–7.7). The rates of infant death and neonatal morbidity were lowest at 40 weeks (18.0/10,000 live births, 95% CI, 13.7–23.6) and 39 weeks (637/10,000 live births, 95% CI, 619–654), respectively. The risk of delivery (per 10,000 pregnancies) was higher at 38 weeks of gestation (815, 95% CI, 793–836) compared with the composite risk of expectant management for an additional week (657.7, 95% CI, 640–676); however, at 39 weeks, the risk of delivery was lower (651, 95% CI, 633–670) compared with the composite risk of expectant management for an additional week (750, 95% CI, 720–781).

**CONCLUSION:**

Among patients with chronic hypertension, delivery at 39 weeks of gestation provides the optimal balance between absolute rates of infant death or neonatal morbidity and stillbirth. This finding is consistent for non-Hispanic Black patients with chronic hypertension and for those pregnancies complicated by fetal growth restriction. This is consistent with current practice guidelines.

## Abstract 2

Among pregnant individuals with hypertension, delivery at 39 0/7 weeks of gestation provides the optimal balance between absolute rates of stillbirth, infant death, and neonatal morbidity.

Chronic hypertension complicates 1.5% of pregnancies in the United States, with prevalence rates increasing, especially among non-Hispanic Black patients.^1^ *Chronic hypertension*, defined as hypertension present before pregnancy or before 20 weeks of gestation, traditionally has been established by systolic blood pressure 140 mm Hg or higher or diastolic blood pressure 90 mm Hg or higher.^1^ Pregnant patients with chronic hypertension are at increased risk for adverse fetal and neonatal outcomes, including fetal growth restriction, stillbirth, low birth weight, preterm birth, and perinatal mortality.^1–6^

The goal of delivery timing is to optimize perinatal outcomes by balancing the risks of stillbirth with ongoing pregnancy and those of neonatal morbidity or mortality associated with early-term delivery. Current recommendations advocating for delivery from 37 0/7 to 39 6/7 weeks of gestation are based on limited evidence.^7–9^ The largest U.S.–based retrospective cohort study that compared stillbirth risk with perinatal morbidity–mortality risk was hampered by the inability to exclude the presence of superimposed preeclampsia, which would lead to overestimation of true perinatal risk.^10^ To estimate the optimal delivery timing among pregnant patients with chronic hypertension, we used a risk-assessment strategy in which the risks of delivery (including risk of infant death or neonatal morbidity) were compared with those of expectant management for an additional week (calculated as the rate of stillbirth over that week plus the rate of infant death or neonatal morbidity in the subsequent week).^11^ The objective was to estimate an optimal delivery timing for pregnancies affected by chronic hypertension in a contemporary U.S. population.

## METHODS

In this population-based retrospective cohort study of term births in the United States from 2014 to 2018, we used data obtained from the National Center for Health Statistics Division of Vital Statistics' birth cohort–linked birth–infant death records and fetal death records.^12^ All variables in the records were abstracted from the individual's medical, prenatal, and delivery records in a standardized fashion and recorded on the newest revision of the U.S. Standard Certificate of Live Birth and U.S. Standard Report of Fetal Deaths.^13,14^ The National Center for Health Statistics natality and mortality files are produced annually under the Vital Statistics Cooperative program and then linked so that information on the infant death certificate is linked to that on the birth certificate for each infant younger than age 1 year.^12^ The data provided by states are reviewed and edited by the National Center for Health Statistics, are coded uniformly, pass quality control standards, and form the basis of the official U.S. birth and death statistics.^15^ This study conformed to the standards and guidelines outlined in the data user agreement with National Center for Health Statistics. The study is exempt from IRB approval at the University of Cincinnati, Ohio, because the database is deidentified and publicly available and thus does not meet criteria for human subjects research. The primary objective was to estimate the optimal timing of delivery in pregnancies complicated by chronic hypertension, accounting for risk of stillbirth with ongoing pregnancy and risk incurred after birth from neonatal morbidity and infant mortality.

Pregnancies were excluded if delivery occurred before 37 weeks of gestation or at 43 or more weeks or if they were complicated by multifetal gestation, diabetes mellitus (preexisting or gestational), or superimposed preeclampsia–eclampsia, because these comorbidities substantially affect delivery timing recommendations. Pregnancies complicated by fetal congenital anomalies were excluded if the anomaly was reported on the natality file or was the cause of death in either the mortality or fetal death file based on the International Classification of Diseases, 10th Revision codes. Beginning with the 2014 fetal death data files, the congenital anomalies variables were dropped in favor of reporting an anomaly if it was the initiating cause of death.^12^ Thus, only anomalies resulting in fetal death were excluded from the stillbirth cohort, whereas all anomalies were excluded in the live birth cohort. Causes of infant death taken from the International Classification of Diseases, 10th Revision codes on death certificates were grouped into large thematic categories and stratified by gestational age at term.

Gestational age was determined by the best obstetric estimate variable in the birth record, accounting for last menstrual period and ultrasound parameters, as is commonly accepted in clinical practice for gestational age estimation.^16^ Gestational age is reported in completed weeks; ie, 37 completed weeks includes neonates born between 37 0/7 and 37 6/7 weeks. *Small for gestational age* (SGA) was defined as birth weight less than the 10th percentile for gestational age.^17^ *Maternal obesity*, categorized by the World Health Organization as body mass index (BMI, calculated as weight in kilograms divided by height in meters squared) 30 or higher, was calculated from prepregnancy weight and height.^18^ *Neonatal morbidity* was defined as a composite of neonatal intensive care unit admission, assisted ventilation for 6 hours or longer, seizures, and low 5-minute Apgar score (3 or lower). In long-term follow-up studies, each of these events was associated with death or serious disability.^19,20^

The rate of stillbirth at a given gestational age was calculated as the number of stillbirths at that gestational age in weeks per 10,000 ongoing pregnancies. For each completed week of gestation between 37 and 42 weeks, the gestational age–specific rates of infant mortality, of neonatal morbidity, and of infant death or neonatal morbidity were calculated as the number of infants born at this gestational age with either a mortality or morbidity per 10,000 live births at the same gestational age. *Infant death* was defined as death within the first year of life; *neonatal death* was defined as death within the first 30 days of life. Infant death was chosen in lieu of neonatal death because it varies with gestational age at term, shares similar risk factors with stillbirth, and captures the complications of perinatal risk more comprehensively in the era of improved neonatal intensive care.^21,22^ A neonatal morbidity resulting in an infant death was counted as one event. The composite mortality rate with expectant management was calculated as the rate of stillbirth over that week plus the mortality rate experienced by an infant born in the subsequent week of gestation. Similarly, the composite rate of infant death or neonatal morbidity with expectant management was calculated as the rate of stillbirth over that week plus the neonatal morbidity and infant mortality rate in the subsequent week of gestation. The risk of delivery at each week (ie, the rate of infant death or neonatal morbidity) was compared with the risk of expectant management (ie, the rate of stillbirth over that week plus rate of infant death or neonatal morbidity in the subsequent week).

Because non-Hispanic Black patients with chronic hypertension experience a significantly higher burden of stillbirths and infant deaths compared with non-Hispanic White patients with chronic hypertension,^3,23^ a subgroup analysis of this population was performed with the same methodology (risk of delivery vs expectant management for 1 week). An additional subgroup analysis examined optimal delivery timing in pregnant patients with chronic hypertension with the additional complication of fetal growth restriction,^3,5^ as inferred from neonatal birth weight meeting SGA criteria, because fetal growth restriction is on the causal pathway to stillbirth among pregnant patients with chronic hypertension.

To estimate the optimal delivery timing among pregnant patients with chronic hypertension, the risk of delivery at each week (ie, the rate of infant death or neonatal morbidity) was compared with the risk of expectant management (ie, the rate of stillbirth over that week plus the rate of infant death or neonatal morbidity in the subsequent week) for an additional week.

Statistical analyses were performed with Stata 15. Rates, relative risks, and 95% CIs were presented for the outcomes of stillbirth, neonatal death, neonatal morbidity, and infant death. Logistic regression was used to determine the relative risk of the outcome measures (stillbirth, infant death, neonatal morbidity, infant death or neonatal morbidity, and expectant management risks) associated with delivery at each week of gestation. The Pearson correlation coefficient (*R*) was used to measure the strength of linear relationships. Proportions of independent variables were compared with the χ^2^ test, and means were compared with analysis of variance. The number needed to deliver at each gestational age was calculated by taking the reciprocal of the absolute risk difference between delivery and expectant management. Statistical significance was assigned to either a value of *P*<.05 or nonoverlapping 95% CI.

## RESULTS

**Fig. 1.**
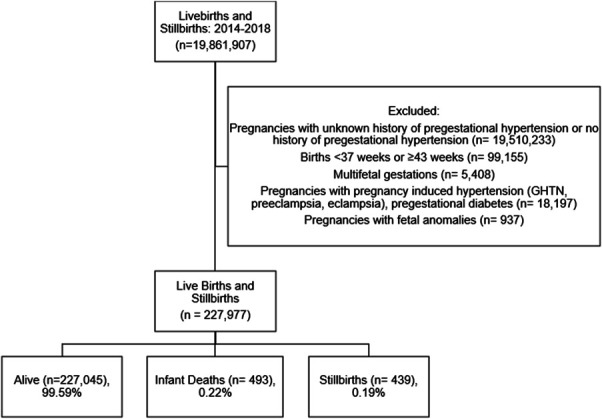
Flow diagram of study population. GHTN, gestational hypertension.

Chronic hypertension complicated 351,674 pregnancies in the United States between 2014 and 2018, corresponding to a prevalence rate of 1.8%. We excluded births occurring before 37 weeks of gestation or at 43 or more weeks (n=99,155), multifetal gestations (n=5,408), and pregnancies complicated by fetal anomalies (n=937), preeclampsia, eclampsia, or pregestational diabetes (n=18,197) (Fig. 1). Of the 227,977 term singleton deliveries in patients with chronic hypertension, the rate of stillbirth was 0.19% (n=439) and the rate of infant death was 0.22% (n=493).

*Hamilton. Delivery Timing and Chronic Hypertension. O&G Open 2024*.

Baseline pregnancy characteristics (Table 1) reveal that the stillbirth and infant death cohorts had higher rates of specific patient characteristics, including young parturient age (younger than 18 years), less than a high school diploma, no prenatal care, and tobacco use. Non-Hispanic Black patients with chronic hypertension had a stillbirth rate of 0.27% and infant death rate of 0.33% with compared with non-Hispanic White patients with chronic hypertension (0.15% and 0.18%, respectively). The rate of neonates with SGA birth weight was higher in pregnancies complicated by stillbirth and infant death (40.1% and 27.6%, respectively) compared with pregnancies that resulted in a living infant at 1 year of life (14.2%).

**Table 1.** Baseline Pregnancy Characteristics Among Patients

Characteristics	Stillbirth (n=439)	Infant Death (n=493)	Alive (n=227,045)
Ethnicity and race			
Hispanic	45 (10.2)	49 (9.9)	32,465 (14.2)
Non-Hispanic Black	163 (37.1)	200 (40.6)	60,337 (26.6)
Non-Hispanic White	182 (42.5)	208 (42.2)	118,010 (51.2)
Other	41 (9.3)	33 (6.7)	14,789 (6.5)
Age group (y)			
Younger than 18	—*	—*	913 (0.4)
18–34	282 (64.2)	382 (77.5)	159,966 (70.2)
35 or older	154 (35.1)	108 (21.9)	66,166 (29.0)
Nulliparity	124 (28.3)	88 (17.9)	60,511 (26.7)
Less than high school diploma	73 (16.7)	101 (20.5)	24,663 (10.8)
Married	NA†	180 (36.5)	128,097 (56.4)
WIC enrollment	139 (31.7)	269 (54.5)	94,082 (41.4)
Prenatal care			
No prenatal care	40 (8.1)	20 (4.1)	2,464 (1.1)
Late initiation (more than 20 wk of gestation)	101 (11.2)	493 (22.5)	24,797 (10.9)
Tobacco use	67 (15.2)	112 (22.7)	20,441 (9.0)
Maternal BMI (kg/m^2^)	35.5±9.4	34.1±9.7	33.4±8.9
Obesity (BMI 30 or higher)	262 (59.6)	282 (57.2)	133,067 (58.6)
Gestational age (wk)	38.4±1.4	38.5±1.3	38.6±1.2
Birth weight (g)	2,868±977	3,047±596	3,256±508
SGA birth weight (less than the 10^th^ percentile)	178 (40.1)	136 (27.6)	32,185 (14.2)

NA, not available; WIC, Special Supplemental Nutrition Program for Women, Infants, and Children; BMI, body mass index; SGA, small for gestational age.

Data are n (%) or mean±SD.

* Count suppressed when fewer than 10 events present.

† Not recorded in the fetal death data.

**Fig. 2.**
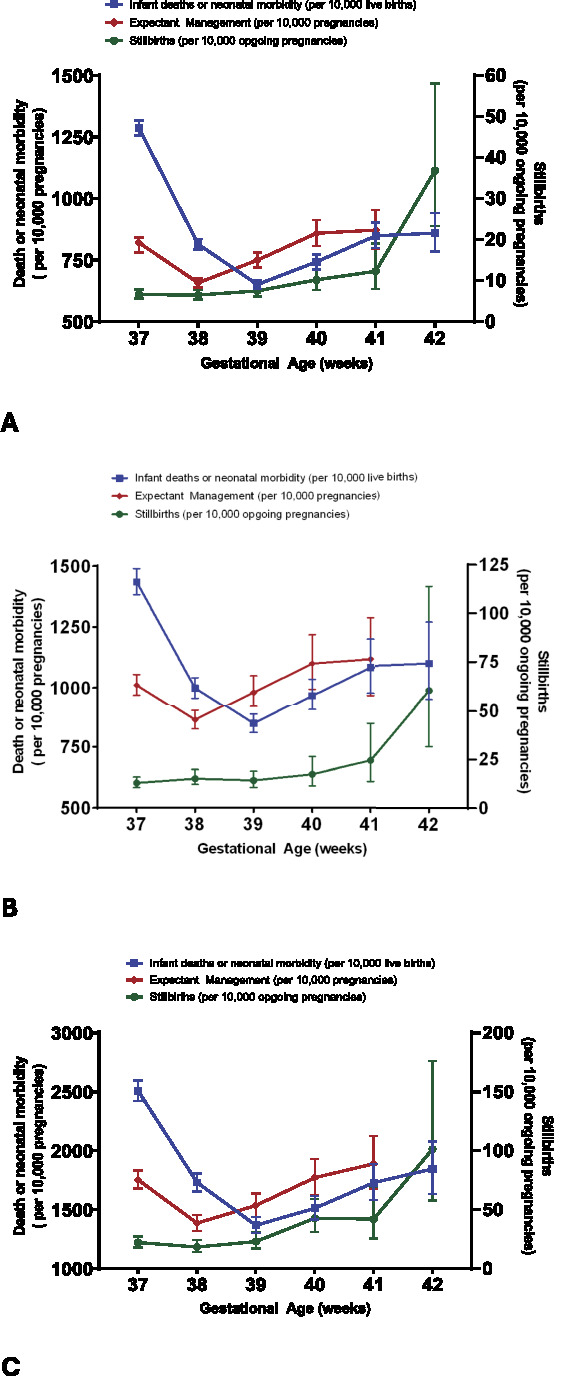
Comparison of the risk of delivery (represented by rate of infant death or neonatal morbidity) with the risk of expectant management for 1 additional week (represented by rate of stillbirth plus rate of infant death or neonatal morbidity at subsequent week) for each week at term among pregnant patients with chronic hypertension (**A**), non-Hispanic Black patients with chronic hypertension (**B**) and pregnant patients with chronic hypertension and fetal growth restriction (**C**). The stillbirth rate is also displayed for reference (*scale to right of graph*).

Table 2 provides the count of ongoing pregnancies, rates of stillbirth (per 10,000 ongoing pregnancies), infant death, neonatal morbidity, and combined infant death or neonatal morbidity (per 10,000 live births) and composite rates of mortality and of infant death or neonatal morbidity with expectant management at each week of term gestation, along with the 95% CIs. The rate of stillbirth increased with gestational age, from 6.6 (95% CI, 5.7–7.8) at 37 weeks of gestation to 36.8 (95% CI, 23.3–58.1) at 42 weeks per 10,000 ongoing pregnancies. The infant mortality rate followed a parabolic curve, with higher rates at 37 and 42 weeks of gestation compared with 38, 39, and 40 weeks (Fig. 2A). The infant mortality risk for expectant management for an additional week was 25.0 (95% CI, 21–28.8) at 38 weeks of gestation and 25.5 (95% CI, 20.3–32.0) at 39 weeks per 10,000 ongoing pregnancies. The observed rates of neonatal morbidity (637, 95% CI, 619–654) and composite of infant death or neonatal morbidity (651, 95% CI, 633–670) per 10,000 ongoing pregnancies were statistically the lowest at 39 weeks of gestation (Table 2).

**Table 2.** Risk of Stillbirth, Infant Death, Neonatal Morbidity, and Expectant Management by Gestational Age Among Women With Chronic Hypertension

Gestational Age (wk)	Ongoing Pregnancies (n)	Stillbirth Rate/10,000 Ongoing Pregnancies	Rate of Infant Deaths/10,000 Live Births	Composite Mortality Rate With Expectant Management for 1 wk/10,000 Pregnancies*	Rate of Neonatal Morbidity/10,000 Live Births†	Rate of Infant Deaths or Neonatal Morbidity/10,000 Live Births	Composite Rate of Infant Death or Neonatal Morbidity With Expectant Management for 1 wk/10,000 Pregnancies‡
37	227,977	6.6 (5.7–7.8)	27.5 (23.2–32.6)	28.9 (25.0–33.4)	1,269 (1,239–1,299)	1,287 (1,257–1,317)	821.2 (780.0–843.0)
38	179,847	6.5 (5.4–7.7)	22.3 (18.9–26.2)	25.0 (21.2–28.8)	797 (776–818)	815 (793–836)	657.7 (639.8–676.0)
39	117,294	7.5 (6.1–9.2)	18.5 (15.6–21.9)	25.5 (20.3–32.0)	637 (619–654)	651 (633–670)	750.2 (720.4–781.2)
40	45,115	10.2 (7.7–14.6)	18.0 (13.7–23.6)	31.5 (22.7–43.6)	730 (700–760)	743 (713–773)	859.6 (806.9–912.7)
41	16,202	12.3 (8.0–19.1)	21.3 (14.3–31.6)	39.0 (25.0–60.8)	835 (786–888)	849 (799–902)	872.0 (796.0–954.5)
42	4,892	36.8 (23.3–58.1)	26.7 (15.6–45.6)	—	843 (768–925)	860 (784–942)	—

Data in parentheses are 95% CI.

* Composite mortality risk with expectant management for 1 week=risk of stillbirth at this gestational age+risk of infant death at the next gestational age week.

† Neonatal morbidity: composite of neonatal intensive care unit admission, ventilatory support for 6 hours or longer, seizures, and low 5-minute Apgar score (3 or lower).

‡ Composite risk of infant death or neonatal morbidity with expectant management for 1 week=risk of stillbirth at this gestational age+risk of infant death or neonatal morbidity at the next gestational age week.

*Hamilton. Delivery Timing and Chronic Hypertension. O&G Open 2024*.

For the primary objective of determining the optimal timing of delivery, the risk of delivery at each week (ie, the rate of infant death or neonatal morbidity) was compared with the risk of expectant management (ie, the rate of stillbirth over that week plus the rate of infant death or neonatal morbidity in the subsequent week). The risk of delivery (per 10,000 pregnancies) was higher at 38 weeks of gestation (815, 95% CI, 793–836) compared with the composite risk of expectant management for an additional week (657.7, 95% CI, 640–676) (Table 2), favoring continuation of pregnancy. However, at 39 weeks of gestation, the risk of delivery was lower (651, 95% CI, 633–670) compared with the composite risk of expectant management for an additional week (750, 95% CI, 720–781). The risk of expectant management was higher than the risk of delivery at 39 weeks of gestation (RR 1.15, 95% CI, 1.10–1.21) and 40 weeks (RR 1.16, 95% CI, 1.08–1.24) (Table 3). The number needed to deliver among ongoing pregnancies at 39 weeks of gestation to prevent a single excess case of neonatal morbidity or mortality was 101 patients (95% CI, 74–156), and the number needed to deliver at 40 weeks was 86 (95% CI, 56–173) compared with expectant management for an additional week.

**Table 3.** Comparative Risks of Stillbirth, Infant Death, and Expectant Management Among Women With Chronic Hypertension

Gestational Age (wk)	Stillbirth Risk	Infant Death Risk	Risk of Infant Death or Serious Neonatal Morbidity	Expectant Management Mortality Risk	Expectant Management Risk*	RR of Expectant Management Compared With Delivery
37	Referent	1.49 (1.17–1.90)	1.98 (1.91–2.05)	Referent	Referent	0.64 (0.62–0.66)
38	0.97 (0.76–1.24)	1.21 (0.95–1.53)	1.25 (1.20–1.30)	0.86 (0.70–1.06)	0.80 (0.77–0.83)	0.81 (0.78–0.84)
39	1.13 (0.87–1.47)	Ref	Ref	0.88 (0.67–1.16)	0.91 (0.87–0.96)	1.15 (1.10–1.21)
40	1.54 (1.11–2.14)	0.98 (0.71–1.35)	1.14 (1.09–1.20)	1.09 (0.76–1.56)	1.05 (0.98–1.12)	1.16 (1.08–1.24)
41	1.86 (1.17–2.97)	1.15 (0.75–1.78)	1.30 (1.22–1.39)	1.35 (0.84–2.16)	1.06 (0.97–1.17)	1.03 (0.92–1.15)
42	5.56 (3.41–9.05)	1.45 (0.82–2.55)	1.32 (1.2–1.45)	—	—	—

RR, relative risk; Ref, referent.

Data in parentheses are 95% CI.

* Expectant management risk=risk of stillbirth at this gestational age+risk of infant death or serious neonatal morbidity at the next gestational age week.

The subgroup analyses of non-Hispanic Black patients and pregnancies complicated by fetal growth restriction also demonstrated statistically lowest risk with delivery at 39 weeks of gestation (Tables 4–7), despite overall higher rates of stillbirth, infant death, and neonatal morbidity (Fig. 2B and C). The number needed to deliver to prevent a case of neonatal morbidity or mortality among the non-Hispanic Black cohort was 74 patients (95% CI, 47–168) at 39 weeks of gestation. Among pregnancies complicated by fetal growth restriction and chronic hypertension, the absolute rates of composite infant death and neonatal morbidity were approximately double the rates observed in the original cohort group (39 weeks of gestation: 11,536.0 [95% CI, 1,439.3–1,638.7] vs 750.2 [95% CI, 720.4–781.2]). Thus, in this subgroup, 59 patients (95% CI, 34–204) needed to deliver at 39 weeks of gestation to prevent a single excess case of neonatal morbidity or mortality compared with expectant management for an additional week.

**Table 4.** Risk of Stillbirth, Infant Death, Neonatal Morbidity, and Expectant Management Among Non-Hispanic Black Women With Chronic Hypertension

Gestational Age (wk)	Ongoing Pregnancies (n)	Stillbirth Rate/10,000 Ongoing Pregnancies	Rate of Infant Death/10,000 Live Births	Composite Mortality Rate With Expectant Management for 1 wk/10,000*	Rate of Neonatal Morbidity/10,000 Live Births†	Rate of Infant Death or Neonatal Morbidity/10,000	Composite Rate of Infant Death or Neonatal Morbidity With Expectant Management for 1 wk/10,000‡
37	67,785	12.7 (10.3–15.7)	41.0 (32.3–52.0)	47.7 (38.8–58.5)	1,412 (1,359–1,466)	1,435 (1,382–1,490)	1,011.3 (969.1–1,055.1)
38	51,233	14.9 (11.9–19.6)	35.0 (27.5–44.5)	44.5 (36.2–54.8)	971 (929–1,014)	999 (957–1,042)	860.5 (822.3–900.3)
39	32,215	14.0 (10.4–18.7)	29.7 (23.0–38.3)	34.8 (23.9–50.7)	823 (785–862)	846 (808–885)	980.7 (916.2–1,049.3)
40	12,245	17.2 (11.2–26.2)	20.9 (12.8–33.8)	77.2 (51.5–115.4)	952 (889–1,020)	967 (903–1,035)	1,100.8 (993.8–1,217.9)
41	4,554	24.2 (13.5–43.2)	60.0 (38.0–94.7)	64.4 (34.6–119.6)	1,050 (946–1,65)	1,084 (977–1,200)	1,118.1 (968.0–1,288.2)
42	1,496	60.2 (31.7–113.9)	40.3 (18.5–87.6)	—	1,087 (939–1,255)	1,094 (945–1,263)	—

Data in parentheses are 95% CI.

* Composite mortality risk with expectant management for 1 week=risk of stillbirth at this gestational age+risk of infant death at the next gestational age week.

† Neonatal morbidity: composite of neonatal intensive care unit admission, ventilatory support for 6 hours or longer, seizures, and low 5-minute Apgar score (3 or lower).

‡ Composite risk of infant death or neonatal morbidity with expectant management for 1 week=risk of stillbirth at this gestational age+risk of infant death or neonatal morbidity at the next gestational age week.

**Table 5.** Comparative Risks of Stillbirth, Infant Death, and Expectant Management Among Non-Hispanic Black Patients With Chronic Hypertension

Gestational Age (wk)	Stillbirth Risk	Infant Death Risk	Risk of Infant Death or Serious Neonatal Morbidity	Expectant Management Mortality Risk	Expectant Management Risk*	RR of Expectant Management Compared With Delivery
37	Referent	1.38 (0.97–1.96)	1.69 (1.59–1.79)	Referent	Referent	0.71 (0.67–0.75)
38	1.17 (0.86–1.59)	1.17 (0.83–1.67)	1.18 (1.11–1.26)	0.93 (0.70–1.25)	0.85 (0.80–0.91)	0.86 (0.81–0.92)
39	1.10 (0.77–1.58)	Ref	Ref	0.73 (0.48–1.12)	0.97 (0.90–1.05)	1.16 (1.07–1.26)
40	1.35 (0.84–2.18)	0.70 (0.40–1.22)	1.14 (1.05–1.24)	1.62 (1.03–2.56)	1.09 (0.98–1.22)	1.14 (1.00–1.29)
41	1.91 (1.02–3.56)	2.02 (1.19–3.42)	1.28 (1.15–1.43)	1.35 (0.70–2.62)	1.11 (0.95–1.28)	1.03 (0.87–1.23)
42	4.74 (2.39–9.40)	1.35 (0.59–3.14)	1.29 (1.11–1.51)	—	—	—

RR, relative risk; Ref, referent.

Data in parentheses are 95% CI.

* Expectant management risk=risk of stillbirth at this gestational age+risk of infant death or serious neonatal morbidity at the next gestational age week.

**Table 6.** Risk of Stillbirth, Infant Death, Neonatal Morbidity, and Expectant Management Among Women With Chronic Hypertension and Fetal Growth Restriction

Gestational Age (wk)	Ongoing Pregnancies (n)	Stillbirth Rate/10,000 Ongoing Pregnancies	Rate of Infant Death/10,000 Live Births	Composite Mortality Risk With Expectant Management for 1 wk/10,000 Pregnancies*	Rate of Neonatal Morbidity/10,000 Live Births†	Rate of Infant Death or Neonatal Morbidity/10,000 Live Births	Composite Risk of Infant Death or Neonatal Morbidity with Expectant Management for 1 wk/10,000 Pregnancies‡
37	37,473	22.2 (17.9–27.5)	55.9 (42.5–73.4)	63.5 (49.4–81.7)	2,476 (2,389–2,566)	2,509 (2,421–2,599)	1,754.4 (1,678.9–1,832.4)
38	28,208	18.4 (14.1–24.2)	41.3 (30.3–46.5)	54.5 (41.9–71.0)	1,698 (1,624–1,775)	1,732 (1,657–1,810)	1,386.7 (1,320.2–1,455.9)
39	18,684	23.0 (17.1–30.8)	36.1 (26.1–50.0)	56.9 (39.5–81.7)	1,343 (1,278–1,412)	1,368 (1,302–1,437)	1,536.0 (1,439.3–1,638.7)
40	8,636	42.8 (31.1–59.0)	33.9 (21.2–54.2)	102.8 (69.0–152.0)	1,499 (1,403–1,601)	1,513 (1,417–1,615)	1,772.7 (1,623.5–1,932.5)
41	3,560	42.1 (25.6–69.4)	60.0 (35.6–100.0)	110.9 (64.9–189.2)	1,700 (1,553–1,857)	1,730 (1,582–1,888)	1,889.2 (1,674.5–2,124.2)
42	1,184	101.4 (58.1–176.3)	68.7 (34.9–135.0)	—	1,821 (1,610–2,054)	1,847 (1,635–2,080)	—

Data in parentheses are 95% CI.

* Composite mortality risk with expectant management for 1 week=risk of stillbirth at this gestational age+risk of infant death at the next gestational age week.

† Neonatal morbidity: composite of neonatal intensive care unit admission, ventilatory support for 6 hours or longer, seizures, and low 5-minute Apgar score (3 or lower).

‡ Composite risk of infant death or neonatal morbidity with expectant management for 1 week=risk of stillbirth at this gestational age+risk of infant death or neonatal morbidity at the next gestational age week.

**Table 7.** Comparative Risks of Stillbirth, Infant Death, and Expectant Management by Gestational Age Among Pregnant Patients With Chronic Hypertension and Fetal Growth Restriction

Gestational Age (wk)	Stillbirth Risk	Infant Death Risk	Risk of Infant Death or Serious Neonatal Morbidity	Expectant Management Mortality Risk	Expectant Management Risk*	RR of Expectant Management Compared With Delivery
37	Referent	1.55 (1.01–2.37)	Ref	Ref	Ref	0.70 (0.67–0.74)
38	0.83 (0.59–1.18)	1.15 (0.73–1.80)	0.69 (0.65–0.70)	0.86 (0.60–1.24)	0.79 (0.74–0.84)	0.80 (0.75–0.86)
39	1.04 (0.72–1.50)	Referent	0.55 (0.51–0.58)	0.90 (0.57–1.40)	0.88 (0.81–0.95)	1.12 (1.04–1.22)
40	1.93 (1.32–2.85)	0.94 (0.53–1.67)	0.60 (0.56–0.65)	1.61 (1.01–2.59)	1.01 (0.92–1.11)	1.17 (1.05–1.31)
41	1.90 (1.10–3.29)	1.65 (0.89–3.06)	0.69 (0.63–0.76)	1.75 (0.96–3.18)	1.08 (0.95–1.22)	1.09 (0.94–1.27)
42	4.58 (2.50–8.36)	1.90 (0.89–4.09)	0.74 (0.65–0.83)	—	—	—

RR, relative risk; Ref, referent.

Data in parentheses are 95% CI.

* Expectant management risk=risk of stillbirth at this gestational age+risk of infant death or serious neonatal morbidity at the next gestational age week.

To better understand the causes of infant mortality in a cohort of term pregnancies complicated by chronic hypertension, the thematically categorized causes of infant death were stratified by gestational age (Table 8). The two most common causes of death at term are sudden infant death syndrome (23.7%) and accident or trauma (21.9%). The cause of death related to labor and delivery accounts for a small portion of total infant deaths (5.7%); however, the rate increases significantly with gestational age past 38 weeks (*R*=0.93). With advancing gestational age, infection as the cause of infant death decreased (*R*=0.91) and pulmonary causes increased (*R*=0.91).

**Table 8.** Causes of Infant Death by Gestational Age, 37–43 Weeks, Among Women With Chronic Hypertension

Cause of Infant Death	Gestational Age (wk)						Total
	37	38	39	40	41	42	
SIDS	25 (18.9)	35 (25.2)	32 (24.1)	15 (28.9)	—*	—*	117 (23.7)
Accident or trauma	35 (26.5)	31 (22.3)	32 (24.1)	—*	—*	—*	108 (21.9)
Related to labor and delivery	—*	—*	—*	—*	—*	—*	28 (5.7)
Infection	10 (7.6)	—*	10 (7.5)	—*	—*	—*	30 (6.1)
Pulmonary	—*	—*	—*	—*	—*	—*	15 (3.0)
Cardiac	12 (9.1)	—*	—*	—*	—*	—*	28 (5.7)
Neoplasm	—*	—*	—*	—*	—*	—*	10 (2.0)
Other	35 (26.5)	43 (30.9)	43 (29.1)	15 (10.1)	—*	—*	148 (30.0)
Missing	—*	—*	—*	—*	—*	—*	—*
Total	132	139	133	52	24	13	493

SIDS, sudden infant death syndrome.

Data are n (%) or n.

* Count suppressed when fewer than 10 events present.

## DISCUSSION

In this population-based study of patients with chronic hypertension with singleton pregnancies, delivery at 39 weeks of gestation optimally balanced the lowest absolute rates of stillbirth and infant death or neonatal morbidity. Expectant management was favored at 38 0/7–38 6/7 weeks of gestation but not at 39 0/7–39 6/7 weeks in terms of lower overall risk of stillbirth and neonatal morbidity or infant death. This finding was consistent among the cohort of non-Hispanic Black patients with chronic hypertension and for those pregnancies complicated by fetal growth restriction. This finding is consistent with and similar to current practice guidelines in the United States.^24^

The American College of Obstetricians and Gynecologists’ Practice Bulletin “Chronic Hypertension in Pregnancy” advocates for delivery between 37 0/7 and 39 6/7 weeks of gestation on the basis of a limited body of evidence.^24^ In 1983, Sibai et al^8^ observed in a small (n=211) cohort of patients with chronic hypertension that most of the perinatal deaths (four of five) occurred among patients with superimposed preeclampsia, suggesting that perinatal outcomes were similar among patients with uncomplicated hypertensive pregnancies compared with those without chronic hypertension. Similarly, Hutcheon et al^10^ published a retrospective cohort study using National Center for Health Statistics data from 1995 to 2005 (n=171,669) to compare composite neonatal morbidity, mortality, and stillbirth rates between 36 and 41 weeks of gestation and found that delaying delivery until 38 or 39 weeks was associated with substantially lower neonatal morbidity and mortality with minimal effect on stillbirth risk among patients with chronic hypertension. In contrast, our study was based on more contemporaneous National Center for Health Statistics data (2014–2018), allowing the exclusion of patients with superimposed preeclampsia and thus a more realistic estimate of stillbirth risk among patients with uncomplicated chronic hypertension with ongoing pregnancies. The presence of preeclampsia in this at-risk cohort would theoretically overestimate stillbirth risk and bias delivery timing toward earlier gestational ages given the associated risk between adverse fetal and neonatal outcomes and preeclampsia.^24,25^ As predicted, our primary analysis found a slightly delayed optimal timing for delivery, favoring expectant management at 38 weeks of gestation but not beyond 39 weeks. In our subgroup analysis including patients who developed superimposed preeclampsia with expectant management, we excluded patients with preeclampsia from the comparison cohort (risk of delivery) to better estimate both the risk of developing preeclampsia with expectant management and the fetal and neonatal risks among those affected by preeclampsia. Rates of stillbirth at each week were also found to be lower in our cohort. In addition, for our analyses, we used a novel composite risk estimate of expectant management developed by Rosenstein et al^11^ incorporating stillbirth risk plus risk of infant death in the subsequent week to quantify an objective week-to-week risk. However, we modified this technique by incorporating neonatal morbidity into this estimation.

After the publication by Hutcheon et al,^10^ a 2011 workshop addressing the timing of indicated late-preterm and early-term birth recommended delivery at 38–39 weeks of gestation for patients with chronic hypertension not on medication and 37–39 weeks for those on medication on the basis of limited evidence.^9,20^ This remains the recommendation in the most recent American College of Obstetricians and Gynecologists’ Committee Opinion on medically indicated delivery timing.^26^ A 2018 single-center retrospective cohort study by Harper et al^7^ is the basis for consideration of delivery by 39 weeks of gestation because of the increased maternal risk of developing severe preeclampsia without any additional neonatal benefit. However, this study was insufficiently powered to detect small but clinically significant differences in stillbirth or neonatal morbidity. A recent planned secondary analysis of the CHAP (Chronic Hypertension and Pregnancy) trial found no difference in composite maternal or neonatal outcomes between individuals with hypertension who underwent planned delivery and those who underwent expectant management at 37, 38, or 39 weeks of gestation.^27^ However, neonates in the planned delivery cohort at 37 weeks of gestation had higher rates of respiratory distress syndrome (adjusted odds ratio 2.70), whereas planned delivery at 37 and 38 weeks was associated with neonatal hypoglycemia (adjusted odds ratio 1.82–1.97). These findings, similar to our study, suggest no difference in maternal outcomes but increased risk of neonatal adverse outcomes for patients with mild chronic hypertension among the early-term delivery cohort.

There are several limitations to our study, including an inability to stratify chronic hypertension on the basis of medication use, number of medications, and degree of control. However, the association between medication use for chronic hypertension and adverse perinatal outcomes is not supported by the current literature, as evidenced by a 2018 systematic review and meta-analysis that found no significant difference in stillbirth or adverse perinatal outcomes between those with medicated and patients with unmedicated chronic hypertension.^23^ Yet, optimal delivery timing stratified by severity (ie, medication use) of hypertension remains an important clinical question requiring further assessment. Another limitation of our study is that the exact timing of delivery (weeks and days) is not reported, which would have allowed more accurate understanding of optimal delivery timing. Although only anomalies resulting in fetal death could be excluded from the stillbirth cohort, this amounted to only 0.04% of the overall cohort and would not significantly affect outcome estimates, as was seen in analyses that did not exclude anomalies in patients with chronic hypertension (tables and figures available on request). An additional limitation of our study is that we could not examine the rates of neonatal morbidity and mortality incurred by expectant management in those who developed superimposed preeclampsia with expectant management. The National Center for Health Statistics data are unable to separately identify pregnant patients with superimposed preeclampsia, because the facility instruction sheet forces a choice between chronic hypertension and preeclampsia, but the development of eclampsia is an independent variable. This unavoidable limitation of excluding pregnant patients from the expectant management group who progress to superimposed preeclampsia could affect delivery timing recommendations to a later gestational age. Maternal morbidity and mortality as they affect delivery timing were beyond the scope of this study. Given the importance of maternal outcomes associated with delivery timing, further studies are needed to determine the optimal delivery timing from a maternal perspective, especially beyond 39 weeks of gestation, when risk of severe preeclampsia substantially increases, as evidenced by Harper et al.^7^ In addition, from this data set, we are unable to determine indication for delivery and whether the delivery was planned or unscheduled. Therefore, deliveries occurred in nonrandomized fashion, which is a major limitation of this retrospective analysis. However, no studies have randomized patients with chronic hypertension to early-term compared with full-term delivery to determine optimal delivery timing, and all prior studies on this topic are based on retrospective data. Lastly, because of limitations of birth record data, we are unable to identify, exclude, or statistically adjust for individuals in this study cohort with more than one pregnancy, which may bias the results.

Despite these limitations, this analysis is based on a large, generalizable, and contemporary U.S. population of pregnancies complicated by chronic hypertension, excluding those in patients who developed preeclampsia. In addition, our adoption of the novel approach reported by Rosenstein et al^11^ but modified by the incorporation of composite neonatal morbidity as done with Hutcheon et al^10^ analysis allowed a quantification of comprehensive (stillbirth, infant death, and neonatal morbidity) risk of expectant management compared with risk of delivery. Moreover, the robust size of our cohort allowed the assessment of subgroups, including non-Hispanic Black women and neonates with SGA birth weights.

In this population-based study of patients with chronic hypertension with singleton pregnancies, delivery at 39 weeks of gestation optimally balanced the lowest absolute rates of stillbirth and infant death or neonatal morbidity. To prevent one case of stillbirth, infant death, or neonatal morbidity, an estimated 101 patients with chronic hypertension would need to deliver at 39 weeks of gestation as opposed to 40 weeks. Given the approximately 45,000 patients with chronic hypertension who deliver at term each year in the United States, a policy of delivery at 39 weeks of gestation theoretically would prevent 450 adverse perinatal events per year.
